# Dual Functionality of miRNAs During HIV Infection: From Viral Genome Suppression to Immune Response Modulation

**DOI:** 10.3390/epigenomes10020039

**Published:** 2026-06-05

**Authors:** Anna M. Timofeeva, Kseniya S. Aulova, Georgy A. Nevinsky

**Affiliations:** Knorre Institute of Chemical Biology and Fundamental Medicine, Siberian Branch of the Russian Academy of Sciences, Novosibirsk 630090, Russia

**Keywords:** microRNA, miRNA, HIV-1, immune response, signaling pathways, HIV latency, toll-like receptors, NF-κB, JAK-STAT, diagnostic markers, therapeutic targets

## Abstract

Background/Objectives: As important post-transcriptional and epigenetic regulators of gene expression, miRNAs play a pivotal role in modulating host–virus interactions. While prior reviews have addressed either direct miRNA–HIV genome interactions or miRNA-mediated immune modulation in isolation, the integrated dual functionality of these molecules has not been systematically characterized. This review aimed to comprehensively explore how miRNAs that target the HIV-1 genome simultaneously modulate key innate and adaptive host immune signaling pathways. The conceptual novelty of this study is determined not by the identification of previously unknown miRNA-target gene pairs, but by the systemic integration of two regulatory levels (direct inhibition of the viral genome and modulation of the host cell immune signaling pathways) within a unified analytical framework. Such an integrated approach reveals a proviral regulatory network that remains non-obvious when each of these levels is examined separately. Methods: A narrative review was conducted using PubMed, Scopus, Web of Science, and Google Scholar (all years through 2025). In Stage 1, publications reporting experimentally confirmed interactions between host miRNAs and the HIV-1 genome were identified, yielding a curated set of 15 miRNAs. In Stage 2, target genes for each miRNA were retrieved from miRTarBase, TarBase (experimentally validated) and TargetScan 8.0 (in silico predicted). In Stage 3, target genes were manually mapped to key immune signaling pathways (TLR, NF-κB, JAK-STAT). In Stage 4, targeted literature searches were performed for each miRNA–target gene pair to identify direct experimental evidence of interaction. All stages were performed by two independent researchers, with discrepancies resolved by a third. Results: Fifteen host miRNAs with experimentally confirmed binding to the HIV-1 genome were identified, targeting viral genes including *nef*, *pol*, *vpr*, *gag*, *env*, *vif*, and the 3′-UTR. Thirteen of these miRNAs were found to regulate components of major immune pathways. miR-92a-3p, miR-29a/b-3p, miR-150-5p, and miR-125b-5p emerged as the most pleiotropic regulators, simultaneously suppressing TLR signaling (TLR3, TLR7, TLR8, MyD88, TRAF3/6, IRAK1/4), NF-κB components (REL, RELA, NFKB1), JAK-STAT effectors (STAT1–3, STAT5A/B, JAK2), and negative regulators of cytokine signaling (SOCS and PIAS family proteins). miR-133b and miR-196b-5p were found to selectively regulate SOCS/PIAS proteins without involvement in other analyzed pathways, suggesting potential for selective therapeutic targeting. Conclusions: The analyzed miRNAs exhibit functional dualism, acting as direct post-transcriptional suppressors of the HIV-1 genome while simultaneously functioning as epigenetic modulators of host immune signaling. These two modes of action are not independent but together form a conceptual framework of a self-reinforcing proviral regulatory network that, based on the synthesis of published evidence, is proposed to promote viral latency and immune evasion. The identified miRNAs represent promising, albeit complex, targets for novel therapeutic strategies aimed at eliminating latent HIV reservoirs.

## 1. Introduction

MiRNAs and their ability to regulate gene expression were discovered in 1993 [1]. Numerous studies have shown that these molecules play an important role in regulating mRNA expression [2]. MiRNA research has been conducted extensively in the context of cancer [3], and their role has also been established in neurodegenerative [4] and in infectious diseases [5,6]. During viral infection, miRNAs influence the host–virus interaction in various ways. MiRNAs can directly bind to the viral RNA genome and suppress viral replication, and can also regulate specific processes in host cells. MiRNAs are also involved in regulating both the innate and adaptive immune responses to viral infections [7]. Furthermore, a single miRNA is capable of regulating numerous different mRNAs, while a single mRNA can be targeted by several miRNAs simultaneously [8].

HIV-1 continues to be a major global public health challenge. According to UNAIDS, since the start of the epidemic, around 88.4 million people have acquired HIV, and around 42.3 million people have died of AIDS-related illnesses [9]. HIV-1 primarily targets T-helper cells, which are key regulators of the humoral and cellular immune response, and where the majority of viral replication occurs. HIV-1 leads to the progressive loss of CD4+ T-helper cells and also infects other cell types, including macrophages and dendritic cells [10,11]. All these host cells also play a crucial role in innate and adaptive immunity. In addition, immune cells frequently serve as viral reservoirs by harboring transcriptionally inactive proviruses, enabling HIV to maintain a persistent infection and evade both immune surveillance and therapeutic interventions [12]. The resulting viral latency remains one of the primary barriers to achieving an HIV-1 cure, as latently infected cells are invisible to immune surveillance and persist despite current antiretroviral therapy [13]. HIV-1 infection can currently be effectively suppressed by antiretroviral therapy (ART). However, the virus establishes a reservoir in long-lived infected immune cells during the early stage of primary infection, and lifelong ART is required to prevent disease progression [14,15].

Changes in miRNA expression profiles have been widely reported during HIV-1 infection [16,17,18,19]. MiRNAs play a multifaceted role in HIV pathogenesis, functioning as key epigenetic regulators. They not only directly interact with the viral genome but also modulate diverse cellular signaling pathways. A central challenge in HIV-1 research is therefore to define the specific role of individual miRNAs in disease pathogenesis and their effects on host gene expression.

Although inhibition of certain miRNAs is considered a promising strategy for eliminating latent HIV reservoirs [17,20,21], existing studies have largely focused either on direct miRNA-mediated effects on the virus or on the regulation of individual host genes. A systematic analysis of the full spectrum of cellular targets affected during HIV infection is generally lacking. Ensuring the safety of miRNA-based therapeutics requires a comprehensive understanding of all host genes whose expression may be altered by such interventions.

This review aimed to comprehensively investigate how miRNAs that interact with the HIV-1 genome modulate critical innate and adaptive immune pathways. MiRNAs that both directly target the viral genome and contribute to the modulation of host immune pathways were selected. Fifteen miRNAs with experimentally confirmed direct interactions with the HIV-1 genome were filtered, and their roles in regulating immune-related pathways were subsequently characterized.

This analysis reveals a dual functionality of miRNAs: they act as direct post-transcriptional regulators of the HIV-1 genome while simultaneously serving as epigenetic modulators of host immune signaling pathways. These two modes of action are not independent but are integrated into a cohesive regulatory network.

While prior reviews have examined direct miRNA–HIV-1 targeting and immune modulation separately, this work integrates both dimensions into a single analytical framework. Starting from a curated set of 15 miRNAs with experimentally confirmed binding to the HIV-1 genome, we systematically mapped their targets within key innate and adaptive immune signaling pathways (TLR, NF-κB, JAK-STAT). This dual-layer approach (combining direct antiviral activity with host immune modulation) represents the conceptual novelty of this work and distinguishes it from existing reviews. While the dual involvement of miRNAs in both direct viral targeting and host immune modulation has been noted in prior reviews, these analyses have not been integrated within a single analytical framework applied to a defined, experimentally validated miRNA panel. For example, Rashid et al. [22] catalogued miRNAs targeting the HIV-1 genome and host dependency factors separately, without systematic pathway-level mapping. Santiago et al. [23] focused on HIV-induced miRNAome alterations and therapeutic delivery challenges, without defining a curated miRNA panel based on direct genome binding evidence. Mansour et al. [24] described miRNAs as dual regulators in broad terms, but did not map a defined miRNA set to multiple immune signaling cascades simultaneously. The present work addresses this gap by showing, for the first time within a single integrated framework, that the same 15 experimentally validated miRNAs simultaneously suppress viral genome translation and modulate five distinct host immune signaling axes. These two activities are mechanistically coupled in a pattern consistent with a self-reinforcing proviral regulatory network. This network represents a conceptual synthesis derived from published data.

## 2. Results

The HIV-1 genome is approximately 9 kb in size and consists of positive-sense single-stranded RNA (ssRNA) encoding all 15 proteins required for replication and assembly of new virions in infected host cells [25,26]. The *gag* gene encodes the virus’s structural proteins, the matrix (MA), capsid (CA), and nucleocapsid (NC) proteins. The *pol* gene encodes the viral enzymes protease (PR), reverse transcriptase (RT), and integrase (IN). The *env* gene encodes two envelope glycoproteins, gp120-SU (surface unit) and gp41-TM (transmembrane unit). Downstream of *pol* are two regulatory genes, *rev* and *tat*, and four accessory genes, *vif*, *vpr*, *vpu*, and *nef* [27,28], whose products influence the rate of viral particle production.

The *nef* gene product enhances HIV infectivity and downregulates several host cell proteins, including CD4 and major histocompatibility complex I (MHC I) [29]. Nef promotes virus replication and pathogenesis by counteracting host antiviral defenses through multiple immunomodulatory mechanisms [30,31]. HIV-1 strains carrying *nef* deletions are characterized by slower progression to AIDS [32], suggesting that miRNAs targeting the *nef* region have the potential to influence HIV-1 pathogenesis [33]. Ahluwalia et al. demonstrated that miR-29a-3p and miR-29b-3p directly bind the *nef* transcript, suppressing its translation and reducing viral load [33].

Using a combination of bioinformatic and molecular biology approaches, miR-149-5p was shown to target *vpr* [34] and *gag* [35]; miR-133b, miR-138, and miR-378 to target *env* [34,35]; miR-324-5p to target *vif*; and miR-92a to target *pol* [35].

**Figure 1:**
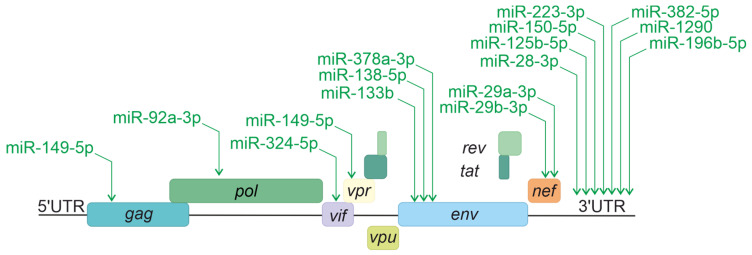
Experimentally validated interactions between human miRNAs and the HIV genome.

Compared to cellular mRNAs, the HIV-1 genome contains a longer 3′-untranslated region (3′-UTR). In addition to its classical role in translation termination and ensuring RNA stability, the 3′-UTR of HIV-1 plays a crucial role in provirus formation and viral packaging [36]. Five miRNAs, miR-28, miR-125b, miR-150, miR-223, and miR-382, target the 3′-UTR and promote proviral latency in resting CD4+ T cells [37]. A dual-luciferase reporter assay was used to experimentally confirm the interaction of miR-1290 and miR-196b with their predicted binding sites in 3′-UTR [17]. The experimentally validated miRNA-HIV genome interaction is summarized in Figure 1 and Table 1.

In total, 15 miRNAs with the ability to directly regulate viral genome translation were identified and selected for further analysis. These miRNAs represent potential therapeutic targets warranting further experimental and clinical validation. Importantly, these miRNAs may regulate not only the viral genome but also a broad range of host biological processes. Next, the immune signaling pathways associated with these miRNAs were analyzed. Using miRTarBase [38], TarBase [39] and TargetScan [40,41], target genes were retrieved for all 15 miRNAs. From the resulting gene list, key genes involved in regulating immune functions were selected. An analysis of the interactions between the selected miRNAs and their corresponding mRNAs was performed. Of the 15 miRNAs, miR-1290 and miR-382-5p had no targets within the analyzed immune pathways and were excluded from subsequent pathway analysis. The remaining 13 miRNAs were examined for their roles in regulating major immune signaling cascades.

The signaling axes analyzed in this review were selected based on the following criteria. First, all pathways are well-established central mediators of innate and adaptive antiviral immunity and are directly implicated in the host response to HIV-1 infection. Second, these pathways are known to be dysregulated during HIV-1 infection and to contribute directly to the establishment and maintenance of viral latency. The SOCS/PIAS regulatory proteins and the T-bet/Eomes transcriptional axis were included as functionally integral components of JAK-STAT regulation and T-cell exhaustion, respectively, given their established roles in HIV reservoir persistence and the failure of immune surveillance over latently infected cells.

### 2.1. Toll-like Receptors

**Figure 2:**
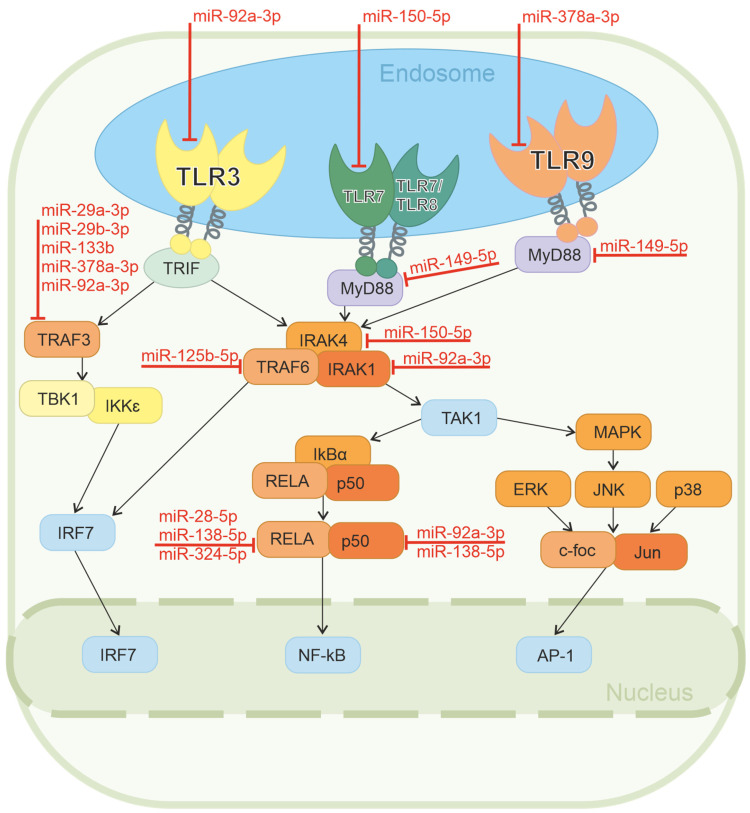
Toll-like receptor signaling pathways. TLR3, TLR7, TLR8, and TLR9 are localized in endosomes. Upon TLR activation through ligand interaction, adaptor molecules are recruited to stimulate downstream signaling pathways, including NF-κB, Activator Protein 1 (AP1), and Interferon Regulatory Factor (IRF). Red arrows indicate the influence of miRNA on individual links of regulatory pathways.

Toll-like receptors (TLRs) are pattern recognition receptors (PRRs) that detect pathogen-associated molecular patterns (PAMPs) and initiate one of the earliest antiviral responses of the innate immune system [42,43]. Endosomal TLRs, such as TLR3, TLR7, TLR8, and TLR9, are particularly important for sensing viral nucleic acids. Most TLRs signal through the MyD88-dependent pathway, which activates NF-κB and drives proinflammatory cytokine production. TLR3 and TLR4 additionally engage the TRIF-dependent pathway, leading to type I interferon induction via interferon regulatory factors (IRFs) [44,45] (Figure 2). Together, these pathways bridge innate and adaptive immune responses [46,47], and miRNA-mediated regulation of their components can therefore directly modulate antiviral defense.

MiR-92a-3p not only targets *pol* [35] but is also involved in regulating TLR3 (Table 2). TLR3 plays a crucial role in antiviral defense by recognizing dsRNA, a common replication intermediate of many viruses [48]. Although HIV is an ssRNA virus, dsRNA-like structures can form during its replication cycle and serve as TLR3 ligands [49]. TLR3 activation induces cellular viral restriction factors, such as interferons (IFNs) type I and type III and IFN-stimulated genes (ISGs) [50]. TLR3 activation significantly inhibits virus infection/replication in human and macaque macrophages [51,52].

The PAR-CLIP (photoactivatable ribonucleoside-enhanced crosslinking and immunoprecipitation) confirmed the interaction of miR-92a-3p with *TLR3* mRNA [55,56] (Table 2). HITS-CLIP (high-throughput sequencing of RNA isolated by crosslinking immunoprecipitation) data demonstrated binding of miR-150-5p to *TLR7* mRNA [53]. MiR-378a-3p is predicted to regulate *TLR8*.

Thus, miRNA-mediated suppression of TLR3, TLR7, and TLR8 may therefore contribute to HIV-1 evasion of early antiviral innate immune signals. This allows the virus to replicate unhindered during the initial stages and also promotes a subsequent transition into a latent state, forming a stable reservoir of infection. These miRNAs can thus be regarded as proviral factors that, by attenuating TLR-driven innate immune sensing, lower the activation threshold required for NF-κB-dependent HIV LTR transcription, reinforcing latency while simultaneously impairing the host’s capacity to clear the reservoir. Notably, several studies have demonstrated that miRNAs themselves can function as TLR ligands [57,58], adding a further layer of complexity to this regulatory circuit.

### 2.2. Intracellular TLR Signaling Pathways

With the exception of TLR3, all TLRs signal through the MyD88-dependent pathway. Upon MyD88 recruitment, a complex forms with IRAK1 and IRAK4 [59,60], leading to TRAF6 activation and downstream engagement of TAK1. TAK1 then activates two parallel pathways: the IKK–NF-κB pathway, which drives proinflammatory gene transcription, and the MAPK pathway, which activates AP-1 via ERK, JNK, and p38 [61,62]. Additionally, TLR7 and TLR9 activation in plasmacytoid dendritic cells (pDCs) triggers IRF7 phosphorylation and IFN-α expression [63]. IRF7 can also be activated via the TRIF-dependent pathway involving TBK1 and IKKε (Figure 2).

MiR-149-5p has been shown to regulate MyD88 and is potentially capable of binding to *TRAF6* mRNA; miR-125b-5p is involved in regulating *TRAF6*; miR-92a-3p targets *IRAK1* and miR-150-5p targets *IRAK4* (Table 3). It has been previously reported that miR-149 directly targets the 3′UTR of *MyD88* and is involved in regulating MyD88 protein expression [64]. MiR-149-5p overexpression has been shown to modulate osteoclast differentiation from bone marrow macrophages through direct inhibition of *TRAF6* expression [65]. The involvement of miR-125b-5p in regulating *TRAF6* has been experimentally confirmed [66,67,68]. According to next-generation sequencing (NGS) data, miR-92a-3p targets *IRAK1* [69].

Five miRNAs are involved in regulating *TRAF3*: miR-29a-3p, miR-29b-3p, miR-133b, miR-378a-3p, and miR-92a-3p (Table 3). The binding of miR-29a-3p and miR-29b-3p to *TRAF3* mRNA has been demonstrated by HITS-CLIP [70,71,72], and confirmed in several independent studies using dual-luciferase reporter assays [74,75,76]. Interaction of miR-92a-3p with TRAF3 mRNA was demonstrated by PAR-CLIP and HITS-CLIP [56,73], and TRAF3 has also been identified as a target of miR-133b [77].

Collectively, the miRNAs (miR-149-5p, miR-125b-5p, miR-92a-3p, miR-150-5p, miR-29a-3p, miR-29b-3p, miR-133b, and miR-378a-3p) target central components of the MyD88-dependent and TRIF-dependent TLR signaling pathways. Because NF-κB binding to the HIV-1 LTR is required for proviral transcription, this coordinated suppression reduces the likelihood of proviral reactivation and thereby contributes to the stability of the latent reservoir.

### 2.3. NF-κB Pathway

NF-κB can be activated through TLR signaling as well as by cytokine receptors, T-cell and B-cell receptors, and other PRRs [78]. It functions as a heterodimeric or homodimeric complex; the most common form is the p65 (RELA)/p50 (NFKB1) heterodimer [79]. Under resting conditions, NF-κB is retained in the cytoplasm by inhibitory IκB proteins. Upon IκB phosphorylation and degradation, NF-κB translocates to the nucleus and regulates the expression of a broad range of target genes.

The level of NF-κB activation at the time of infection is a critical determinant of HIV-1 fate within the cell. Low activity during early infection promotes latent reservoir establishment [80], whereas chronic NF-κB activation in later disease stages drives pathological immune activation, T-cell exhaustion, and viral persistence [81]. HIV-1 viral proteins further modulate this pathway: Tat and Nef enhance NF-κB activation to promote viral replication [82,83], while Vpu and Vpr exert suppressive effects that may facilitate immune evasion and latency establishment [84]. miRNAs with altered expression during HIV-1 infection are also implicated in the fine-tuning of this pathway.

Seven miRNAs (miR-138-5p, miR-150-5p, miR-28-5p, miR-29a-3p, miR-29b-3p, miR-324-5p, miR-92a-3p) have been shown to regulate components of the NF-κB pathway, including *REL*, *RELA* and *NFKB1* (Table 4). CLASH (Cross-linking, Ligation, and Sequencing of Hybrids) demonstrated an interaction between *RELA* mRNA and miR-324-5p [69]. Various CLIP-seq approaches confirmed binding of *REL* mRNA with miR-92a-3p [85], miR-29a-3p and miR-29b-3p [86], and miR-150-5p [87]. An interaction between NFKB1 mRNA and miR-92a-3p was additionally demonstrated by the CLASH [69].

The suppression of NF-κB components by seven of the 15 miRNAs analyzed here can have implications for HIV latency. By targeting RELA, NFKB1, and upstream activators of the NF-κB complex, the identified miRNAs can reduce the nuclear availability of this transcription factor. Because NF-κB is a direct transcriptional activator of HIV-1 replication from the viral LTR, its attenuation by host miRNAs creates a transcriptionally permissive environment for proviral silencing.

### 2.4. JAK, STAT and the JAK-STAT Pathway

**Figure 3:**
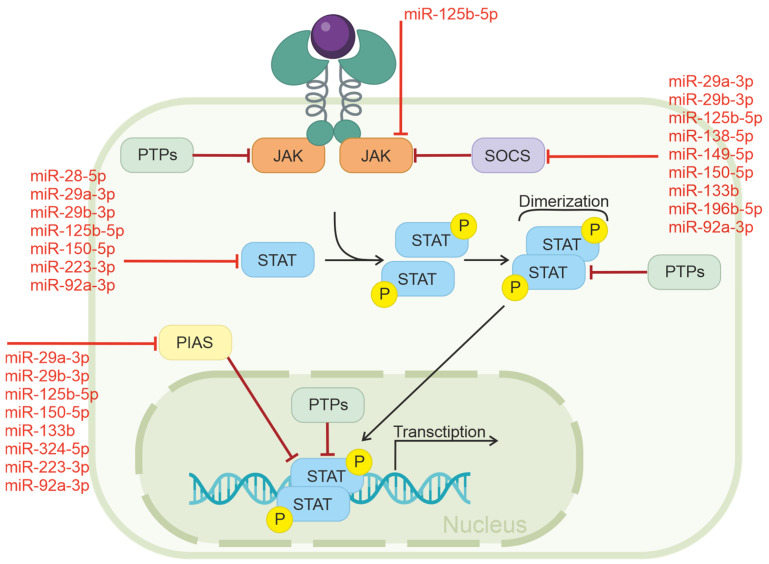
The JAK/STAT signaling cascade. Ligand binding to the receptor leads to the activation of associated Janus kinases (JAK), which phosphorylate STAT proteins. Then the STAT proteins dimerize, translocate to the nucleus, and regulate genes involved in immunity, inflammation, cell proliferation, and apoptosis. Red arrows indicate the influence of miRNAs on specific components of the regulatory pathways.

The JAK-STAT pathway mediates cellular responses to type I interferons (IFN-α/β), type II interferon (IFN-γ), and a broad range of cytokines, hormones, and colony-stimulating factors [89]. The pathway comprises four JAK family kinases (JAK1, JAK2, JAK3, and TYK2) and seven STAT transcription factors (STAT1–4, STAT6, STAT5A, and STAT5B) [90,91]. Upon ligand binding, receptor-associated JAKs phosphorylate STAT proteins, which then dimerize, translocate to the nucleus, and regulate genes involved in immunity, inflammation, cell proliferation, and apoptosis [92] (Figure 3).

The JAK/STAT pathway is one of the key host signaling axes disrupted during HIV-1 infection. HIV-1 suppresses interferon-mediated antiviral responses by blocking JAK/STAT signaling [93,94]. For example, Vif promotes proteasomal degradation of STAT1 and STAT3 [95]. Other HIV proteins modulate STAT transcription factors’ activity [96,97]. HIV-1 stimulates SOCS3 production, which inhibits IFN signaling by disrupting STAT1 and STAT2 phosphorylation [96]. Together, these mechanisms contribute to immune evasion, sustained inflammation, and viral reservoir preservation [98].

*STAT1* is targeted by miR-150-5p and miR-223-3p (Table 5). Luciferase reporter assay has demonstrated that miR-150-5p binds to *STAT1* mRNA and suppresses its expression. Furthermore, *STAT1* expression is inversely correlated with miR-150-5p levels [99]. In cells infected with human T-cell lymphotropic virus type I, *STAT1* expression is co-regulated by miR-150 and miR-223 [100]. Given the established role of miR-150 in lymphocyte development and its altered expression in various diseases [101,102], this regulatory axis warrants further investigation in the context of HIV-1 infection.

MiR-92a-3p regulates *STAT2* (Table 5). The level of the miRNA is elevated in peripheral blood mononuclear cells during HIV-1 infection [119]. STAT2 mediates type I interferon signaling in complex with STAT1 [120]. Thus, miR-92a-3p-mediated suppression of *STAT2* may attenuate the antiviral response.

*STAT3* is regulated by miR-92a-3p, miR-125b-5p, miR-223-3p, miR-29b-3p, and miR-29a-3p (Table 5). STAT3 transduces signals from TLRs, interleukin receptors (e.g., IL-6, IL-10, IL-11), growth factors, and colony-stimulating factors (e.g., granulocyte colony-stimulating factor, G-CSF) [121]. miR-29a-3p targets STAT3, as confirmed in sepsis models [111,112]. MiR-29b-3p negatively regulates *STAT3* [122,123]. A dual-luciferase reporter assay confirmed the interaction between miR-125b-5p and *STAT3* [108,109]. MiR-223-3p directly binds the *STAT3* 3′-UTR, as demonstrated in a diabetic kidney disease model [124]. MiR-92a-3p binding to *STAT3* mRNA was shown by CLASH [69]. In addition to *STAT3*, miR-125b-5p directly targets *JAK2* (Table 5).

*STAT5A* is regulated by miR-223-3p, while *STAT5B* is targeted by miR-28-5p and miR-150-5p (Table 5). It has previously been demonstrated that miR-28-5p targets *STAT5B* [125], which is associated with the regulation of memory T cells and regulatory T cells [126,127], as well as with mast cell activation during skin inflammation [128]. Regulation of *STAT5B* by miR-150-5p has been demonstrated using luciferase reporter assay, qRT-PCR, and Western blot [117].

Collectively, miR-125b-5p, miR-150-5p, miR-223-3p, miR-28-5p, miR-29a-3p, miR-29b-3p, and miR-92a-3p form a complex regulatory network targeting multiple STAT proteins critical for antiviral defense and lymphocyte function. Given that HIV-1 reactivation can proceed via the JAK/STAT5 pathway [129], and that STAT1/STAT2 signaling drives interferon-stimulated gene expression critical for restricting viral spread, the coordinated miRNA-mediated suppression of multiple STAT proteins simultaneously impairs antiviral defense and stabilizes the latent state. This dual effect (reduced immune clearance and attenuated reactivation signaling) makes the STAT-targeting miRNA network a particularly relevant subject for investigation in the context of latency reversal and reservoir elimination strategies.

### 2.5. Regulation of the STAT Pathway

JAK/STAT signaling is tightly regulated by several negative feedback mechanisms, including suppressors of cytokine signaling (SOCS), protein inhibitors of activated STAT (PIAS), protein tyrosine phosphatases (PTPs), and ubiquitin-specific proteases such as USP18. These mechanisms ensure a balance between immune system activation and resolution, thereby maintaining immune homeostasis and preventing pathological inflammation or autoimmunity [130,131,132].

HIV infection is characterized by persistent immune activation, reflected in elevated levels of phosphorylated STAT proteins. It is hypothesized that T cell activation in HIV infection is partly due to the inability of SOCS1 and SOCS3 to adequately control the JAK/STAT signaling pathway. Chronic immune activation disrupts the lymphoid system and promotes HIV replication, as the virus preferentially infects activated cells [133]. It is suggested that modulating SOCS expression or function may represent a potential strategy to counteract immune activation in HIV infection.

The studied miRNAs (miR-125b-5p, miR-133b, miR-138-5p, miR-149-5p, miR-150-5p, miR-196b-5p, miR-29a-3p, miR-29b-3p, miR-324-5p, miR-92a-3p) target SOCS and PIAS family proteins (Table 6). For miR-125b-5p, luciferase assays have demonstrated regulation of *SOCS4* [134] and *PIAS3* [135]. Suppression of *SOCS5* by miR-92a-3p has been shown using qRT-PCR, Western blot, and luciferase reporter assay [136]. Binding of miR-150-5p with *SOCS5* has been demonstrated by PAR-CLIP [137]. An interaction between miR-150-5p and *PIAS2* has been demonstrated by HITS-CLIP [138]. CLASH analysis has shown an interaction between *PIAS4* mRNA and miR-324-5p [69].

JAK/STAT pathway inhibitors are actively discussed in the literature as a promising strategy for HIV-1 treatment in combination with antiretroviral therapy [140,141]. Identification of miRNA-mediated SOCS/PIAS regulation opens a new research direction with direct implications for HIV-1 latency. By fine-tuning the negative feedback on JAK/STAT signaling, these miRNAs adjust the cytokine sensitivity of latently infected cells and thereby modulate the activation threshold at which proviral transcription is triggered. Notably, bioinformatics analysis indicates that miR-133b and miR-196b-5p selectively target SOCS and PIAS family without involvement in the other immune response regulatory pathways considered here, making them promising and potentially selective molecules for therapeutic targeting.

### 2.6. Eomes and T-bet

Eomes and T-bet (TBX21) are members of the T-box family of transcription factors and share significant functional similarity [142]. Both factors regulate the differentiation and function of T cells, NK cells, and select B cell populations during immune responses to infection [143,144,145,146]. In particular, T-bet and Eomes act as central regulators of CD8+ T-cell exhaustion and memory formation, thereby controlling the capacity of cytotoxic T cells to eliminate virus-infected cells [147].

Eomes expression is elevated in HIV-infected patients with viral loads above 50 copies/mL, suggesting a role for this transcription factor in HIV-associated immune activation [148]. In HIV-1 infection, high *EOMES* expression in CD8+ T cells is associated with reduced T-bet expression [149]. Conversely, elevated T-bet expression in memory B cells during HIV infection is associated with unfavorable immunological outcomes, including impaired migration to germinal centers and defective affinity maturation, which compromise effective antiviral humoral immunity [150].

Four miRNAs (miR-29a-3p, miR-29b-3p, miR-92a-3p, miR-28-5p) regulate *EOMES* and *TBX21* (Table 7). *TBX21* suppression by miR-29b has been confirmed by a luciferase assay [151]. The involvement of miR-92a-3p in *EOMES* regulation has been shown by HITS-CLIP [152].

The miRNA-driven shift toward elevated Eomes and reduced T-bet expression thus promotes a dysfunctional CD8+ T-cell phenotype that is unable to eliminate latently infected cells, even upon partial viral reactivation. This functional exhaustion of HIV-specific cytotoxic T lymphocytes constitutes a critical immune evasion mechanism that sustains the latent reservoir independently of direct viral gene expression—and represents a distinct, epigenetically regulated dimension of HIV-1 latency that is amenable to miRNA-based immunotherapeutic intervention.

The miRNAs identified in this review do not act in isolation: many target components across multiple pathways simultaneously, and their combined effect (dampened TLR signaling, attenuated NF-κB and JAK-STAT activation, disrupted SOCS/PIAS feedback, and impaired T-bet-driven cytotoxic immunity) constitutes a coherent. Recognizing this network architecture, rather than treating each pathway independently, is essential for understanding how the virus exploits host miRNA biology to establish a stable reservoir.

## 3. Discussion

MiRNAs play a key role in regulating gene expression during viral infections [7]. While their involvement in oncogenesis has been studied in detail, the interaction of miRNAs with HIV infection remains a topic of active research. This study analyzes the functional duality of host miRNAs in HIV-1 infection: their ability to simultaneously suppress viral replication by directly binding to viral transcripts and modulate host immune signaling pathways.

### 3.1. Functional Dualism of miRNAs in HIV-1 Infection: Direct and Indirect Mechanisms

During acute HIV infection, a subset of CD4+ T cells transitions into a quiescent state permissive for viral latency [153,154,155]. Latently infected cells are invisible to immune surveillance and resistant to current antiretroviral agents, making viral latency the primary barrier to an HIV-1 cure. The latent reservoir is relatively stable over time but not static: sporadic activation of latently infected cells leads to low-level viral production, accounting for the residual viremia detectable in ART-treated individuals.

In the context of HIV latency, host miRNAs can influence the HIV-1 replication cycle at two levels:

(1) Direct interaction with viral transcripts, leading to translational suppression. miRNAs bind directly to viral RNA sequences (including *nef*, *pol*, *vpr*, *gag*, *env*, *vif*, and the 3′-UTR), thereby reducing viral protein synthesis and restricting HIV-1 replication.

(2) Modulation of host immune signaling. This includes regulation of the NF-κB, JAK/STAT, SOCS/PIAS, and the T-bet/Eomes transcriptional axis, as examined in the present work.

**Figure 4:**
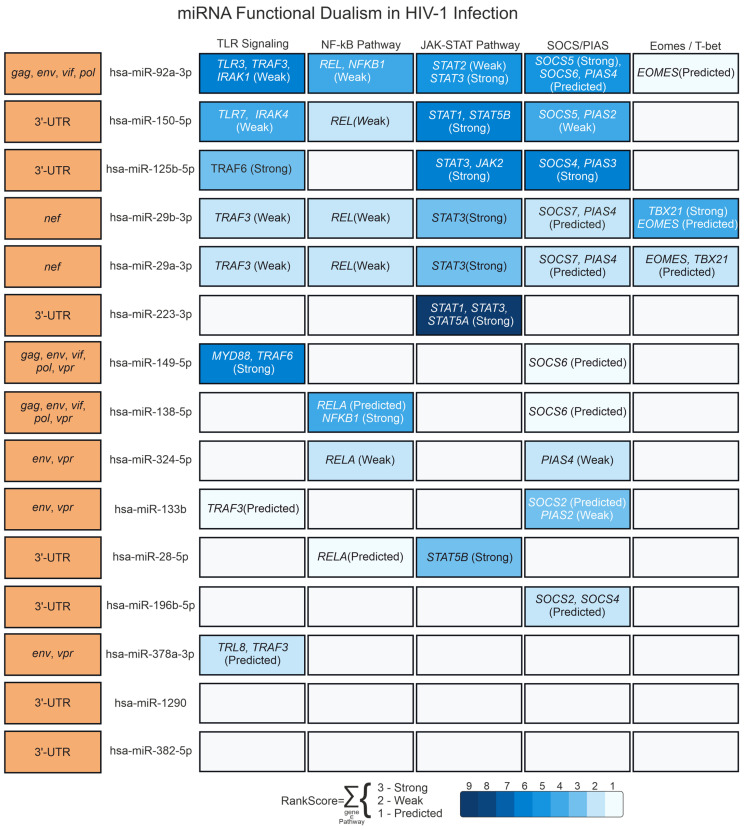
Dual miRNA regulatory network in HIV-1 infection. Left panel: experimentally confirmed interactions between 15 human miRNAs and the HIV-1 genome. Right panel: six categories of immune pathways regulated by the identified miRNAs, with indication of the evidence level of interactions. For each miRNA within a single pathway, an integral score was calculated by summing the weights of all its interactions with genes of that pathway. Weights were assigned depending on the type of experimental confirmation: 3—Functional MTI (Strong, direct functional confirmation), 2—Functional MTI (Weak), 1—predicted interaction (in silico). The integrated model presented in this figure represents a conceptual synthesis based on published experimental and computational data.

The present analysis identified 15 human miRNAs that operate across two levels: direct suppression of viral transcripts and indirect modulation of host immune signaling. Figure 4 provides an integrated visualization of this network: the left panel maps experimentally confirmed miRNA–HIV genome interactions, while the right panel demonstrates miRNA engagement across six immune pathway categories. Together, the two panels illustrate how the same set of miRNAs simultaneously targets the virus and reshapes the host immune environment to favor latency maintenance.

The most direct proviral mechanism involves miRNA binding to HIV-1 genomic RNA (Figure 4, left panel). Studies have demonstrated that a set of cellular miRNAs, including miR-28, miR-125b, miR-150, miR-223, and miR-382, is expressed at higher levels in resting CD4+ T cells than in activated CD4+ T cells. By directly binding to viral RNA, these miRNAs help restrain HIV-1 replication [37]. A similar effect is observed in monocytes, which also express miR-28, miR-150, miR-223, and miR-382. Reduced levels of these miRNAs in monocytes enhance HIV replication, while their expression in macrophages suppresses viral replication [156]. The present review identified experimentally confirmed interactions between miRNAs and internal viral targets. The direct proviral mechanism involves miRNA binding to HIV-1 genomic RNA (Figure 4, left panel).

MiRNAs play an important role in both establishing and sustaining latent HIV-1 infection through modulation of host signaling cascades. Resting CD4+ T cells preserve key transcription initiation factors required for HIV expression, including NF-κB [154,157]. The viral long terminal repeat (LTR) harbors multiple binding sites for this and other cellular transcription factors [158]. Consequently, any stimulus that triggers NF-κB activation can induce expression of the integrated provirus [159]. Specific host miRNAs suppress NF-κB activity, promoting the transcriptional silencing of latent HIV-1. In addition, miRNAs can directly target key upstream regulators of the NF-κB cascade, including TRAF6 and IRAK1, resulting in pathway activation and reinforced viral latency. Seven miRNAs identified in this review target components of the NF-κB complex (RELA, REL, NFKB1) and its upstream activators (TRAF6, IRAK1). Suppression of TRAF6 by miR-149-5p, miR-125b-5p, and targeting of IRAK1 by miR-92a-3p attenuates the MyD88-dependent signal that converges on IKK-mediated IκB degradation. By suppressing this central pro-inflammatory axis, these miRNAs likely serve a critical function in both the induction and long-term preservation of the HIV-1 latent reservoir.

The role of the JAK/STAT pathway in HIV-1 latency is complex and appears to be bidirectional. Cytokine-mediated JAK/STAT activation (primarily via IL-2, IL-7, and IL-15) maintains the homeostasis of CD4+ T cells, thereby promoting reservoir maintenance [160]. By contrast, pharmacological JAK inhibition (ruxolitinib, tofacitinib) blocks reservoir formation and reduces viral production [141]. Specific JAK2 inhibitors demonstrated the ability to reverse HIV latency through IRF7 activation [161], highlighting the context-dependent effects of this pathway. Seven miRNAs characterized in this review (miR-125b-5p, miR-150-5p, miR-223-3p, miR-28-5p, miR-29a-3p, miR-29b-3p, and miR-92a-3p) target STAT1, STAT2, STAT3, STAT5A, and STAT5B. Notably, miR-28-5p targeting of STAT5B and miR-150-5p targeting of STAT5B are particularly relevant given the established role of STAT5 in proviral reactivation. By suppressing multiple STAT proteins simultaneously, these miRNAs reduce the amplitude of cytokine-driven activation signals and thereby lower the probability of stochastic proviral reactivation in resting CD4+ T cells.

SOCS and PIAS proteins constitute the principal negative feedback regulators of JAK/STAT signaling, and impaired SOCS-mediated control has been associated with persistent immune activation in HIV-1 infection [133]. Ten miRNAs (miR-125b-5p, miR-133b, miR-138-5p, miR-149-5p, miR-150-5p, miR-196b-5p, miR-29a-3p, miR-29b-3p, miR-324-5p, miR-92a-3p) identified in this study target SOCS and PIAS. While the majority of these miRNA–SOCS/PIAS interactions have yet to be experimentally confirmed, our findings align with a growing interest in this regulatory layer. Two miRNAs (miR-133b and miR-196b-5p) selectively target SOCS and PIAS family members without involvement in other immune pathways examined here, making them candidates for selective pharmacological exploitation. By dysregulating SOCS/PIAS-mediated feedback, these miRNAs alter the cytokine sensitivity of latently infected cells, effectively shifting the activation threshold at which proviral transcription is triggered.

Effective clearance of the latent reservoir requires functional HIV-specific CD8+ cytotoxic T lymphocytes (CTLs), whose differentiation and effector capacity are governed by the T-bet/Eomes transcriptional axis [147]. Elevated T-bet sustains CTL effector functions, including elimination of HIV-infected cells [150], whereas high Eomes expression during chronic infection is associated with terminal CD8+ T-cell exhaustion and reduced cytotoxic activity [142,143,148,149]. Four miRNAs identified in this review (miR-29a-3p, miR-29b-3p, miR-92a-3p, and miR-28-5p) target *TBX21* (T-bet) and/or *EOMES*. MiRNA-mediated downregulation of T-bet, coupled with maintenance or upregulation of Eomes, fosters a dysfunctional CTL phenotype that is unable to eliminate latently infected cells even upon partial viral reactivation.

Collectively, the five mechanistic arms depicted in Figure 4 are not independent: several miRNAs operate across multiple arms simultaneously. This multi-pathway engagement means that the loss or gain of a single miRNA can have pleiotropic consequences for latency stability.

### 3.2. Translational Perspectives: miRNA-Directed Therapeutic Strategies and Clinical Obstacles

The therapeutic application of specific miRNA mimics and inhibitors constitutes a promising avenue for future investigation and may ultimately complement existing antiretroviral therapy (ART). While current ART regimens effectively suppress active viral replication, they fail to eliminate the latent reservoir. One widely explored approach to reservoir reduction, termed “shock and kill,” seeks to reactivate latent proviruses using agents that induce viral transcription without triggering global T-cell activation [162]. Conversely, the “block and lock” strategy aims to enforce deep latency, thereby preventing spontaneous or induced viral reactivation [163]. In both paradigms, miRNAs offer a means of precise modulation: miRNA mimics that downregulate NF-κB or JAK/STAT signaling could promote latency establishment or maintenance, whereas miRNA inhibitors (antagomirs) could be deployed to facilitate proviral reactivation. Table 8 discusses the main therapeutic strategies that can be used for the miRNAs discussed in this review.

As shown in Table 8, the proposed therapeutic modalities fall into two broad categories aligned with established HIV cure strategies. miR-150-5p, miR-125b-5p, miR-149-5p, miR-138-5p, miR-324-5p, miR-223-3p, and miR-28-5p are candidates for mimic-based “block and lock” approaches, as their validated targets collectively suppress the innate sensing, NF-κB, and JAK/STAT signals required for proviral reactivation. Conversely, miR-92a-3p and miR-29a/b-3p represent antagomir targets for “shock and kill” strategies, where their inhibition would restore immune pathway activity and facilitate reactivation of latent virus for subsequent immune-mediated clearance.

Despite the therapeutic potential of miRNA-based interventions, a number of challenges must be addressed before clinical implementation. Several practical obstacles must be overcome before miRNA-based HIV therapeutics can advance to clinical application. Regarding delivery, the primary challenge is achieving efficient and cell-type-specific delivery of miRNA mimics or antagomirs to the principal HIV reservoir compartments (CD4+ T cells and macrophages). Current delivery platforms under investigation include lipid nanoparticles (LNPs), which have demonstrated efficacy in clinical RNA therapeutics (e.g., mRNA vaccines), and exosome-based delivery systems, which offer natural cell-targeting properties. However, neither platform has yet been validated for miRNA delivery to resting CD4+ T cells in vivo. Off-target effects represent a second major concern. Given that each miRNA can regulate hundreds of target mRNAs, systemic administration of mimics or antagomirs carries a substantial risk of unintended transcriptome-wide effects.

### 3.3. MiRNAs as Diagnostic Tools in HIV Infection

MiRNAs have shown considerable promise as diagnostic biomarkers for HIV infection. HIV infection is associated with pronounced alterations in the expression profiles of specific circulating miRNAs. For example, Thapa et al. reported elevated or reduced serum levels of miR-21, miR-122, and miR-223 in HIV-infected individuals, supporting their potential utility as non-invasive biomarkers [164]. In addition, Narla et al. characterized a distinct expression signature comprising 29 miRNAs in HIV-positive subjects, which may not only shed light on the underlying mechanisms of viral containment and immune regulation but also offer diagnostic or prognostic insights [165]. Other researchers have also identified differentially expressed miRNAs that could be used as biomarkers [166,167].

MiRNAs are also promising as prognostic markers in HIV infection. Their expression levels have been shown to correlate with disease trajectory, immune status, and clinical outcomes. For instance, Moghoofei et al. examined the associations between several miRNAs (miR-29, miR-150, miR-155, miR-223) and virological and immunological parameters in HIV-1-infected individuals, proposing that these miRNAs may influence the pace of clinical progression [168]. Similarly, Swaminathan et al. identified specific miRNAs linked to the advancement of HIV disease toward acquired immunodeficiency syndrome AIDS, highlighting their potential utility in forecasting disease evolution [169]. Such miRNA profiling may facilitate the identification of patients at heightened risk of rapid disease advancement, thus guiding decisions toward more intensive therapy or closer monitoring.

Among the 15 miRNAs characterized in this study, miR-29a-3p, miR-29b-3p, miR-150-5p, and miR-223-3p hold particular diagnostic promise, given that their plasma and PBMC concentrations correlate with key clinical parameters such as viral load, CD4+ T cell counts, and disease stage [119,170]. Combined panels of miRNA biomarkers may provide higher diagnostic accuracy compared to individual markers, and their non-invasive detection in blood plasma makes them attractive candidates for monitoring ART efficacy and estimating the size of the latent reservoir.

### 3.4. Limitations

This study was carried out as a narrative review and is therefore subject to certain methodological limitations that should be considered when interpreting the findings.

o Absence of protocol pre-registration. In contrast to systematic reviews, this study was not registered in PROSPERO or any comparable database. As a result, both the eligibility criteria and the analytic approach were refined during the course of the literature synthesis.
o Publication bias. The set of 15 miRNAs reported to interact with the HIV-1 genome was curated from published experimental evidence. Given that studies reporting positive findings (i.e., confirmed interactions) are substantially more likely to be published than those with null or negative outcomes, the true repertoire of miRNAs capable of engaging the HIV-1 genome may be underrepresented in the current literature.
o Database-associated bias. Target gene identification relied on miRTarBase, TarBase, and TargetScan. Interactions were classified according to the miRTarBase evidence hierarchy: Functional MTI (Strong), Functional MTI (Weak), and predicted (in silico). A substantial proportion of the miRNA–target gene pairs rely exclusively on computational predictions from TargetScan 8.0 and have not been confirmed by direct functional assays such as luciferase reporter experiments, Western blotting, or qRT-PCR with target expression quantification. Accordingly, the biological significance of these predicted interactions should be interpreted with caution. But the absence of experimental confirmation does not constitute evidence against an interaction. It remains possible that a subset of these predicted interactions may eventually receive experimental confirmation. Moreover, the depth of database coverage is highly skewed: extensively characterized miRNAs such as miR-155 and miR-21 are vastly overrepresented relative to understudied species, potentially conveying a misleading impression of their relative biological importance.
o Language bias. The literature search was restricted to English-language publications. As a consequence, relevant studies reported in other languages were excluded, potentially resulting in an incomplete capture of the existing evidence base.
o Absence of formal study quality assessment and risk-of-bias evaluation. No formal assessment of study quality or risk of bias was performed for the primary studies included in this review. The quality of experimental evidence varies considerably across the cited publications, ranging from single-experiment observations in immortalized cell lines to multi-method validations in primary human cells and clinical samples. This heterogeneity in evidence quality was not formally weighted during synthesis. As a consequence, findings supported by a single in vitro experiment are presented alongside those confirmed by multiple independent approaches, without explicit differentiation of their relative evidentiary strength. Readers should interpret conclusions accordingly, with particular caution applied to interactions supported by limited or indirect evidence.
o Subjectivity in manual curation. The assignment of miRNA target genes to specific immune signaling pathways was performed by manual curation. Although this process was conducted independently by two researchers with discrepancies resolved by a third, manual curation remains inherently subjective. Decisions regarding pathway assignment depended on the researchers’ interpretation of the available literature and their judgement regarding the functional relevance of individual gene–pathway relationships. This subjectivity limits the reproducibility of the pathway mapping and may have introduced systematic biases in the selection or exclusion of specific miRNA-target-pathway associations.
o Limitations of causal inference. The regulatory relationships described in this review are based on correlational and experimental evidence from individual studies conducted predominantly in vitro or ex vivo. The review does not establish causal directionality for the described interactions in the context of HIV infection in vivo, and the identified miRNA–target–pathway associations should not be interpreted as proven causal mechanisms in human disease.

Because not all experimentally validated interactions are captured in existing databases, manual curation was employed to expand the list. Furthermore, the lack of experimental confirmation should not be interpreted as evidence against an interaction; it may merely reflect that the interaction has yet to be investigated or reported.

## 4. Methods

This study was conducted as a narrative review.

The following section outlines the literature selection and analysis process; it is not intended as a rigorous systematic review protocol but serves to illustrate the methodological logic underpinning the work.

Stage 1. Identification of host miRNAs interacting with the HIV-1 genome

We searched major databases (PubMed, Scopus, Web of Science, Google Scholar) for English-language publications using the terms “HIV” AND “miRNA”/“microRNA”. The search included all available records published up to 2025. Titles and abstracts were initially screened, and after removing duplicates, full-text articles were reviewed. The inclusion criterion was the presence of experimentally validated interactions between a host miRNA and the HIV-1 genome. This process yielded a final list of 15 human miRNAs with documented evidence of direct HIV-1 genome targeting. The 15 miRNAs retained represent the complete set of human miRNAs for which peer-reviewed experimental evidence of direct HIV-1 genome binding was available in the literature as of 2025. No eligible miRNAs were excluded.

Stage 2. Determining the target genes of the selected miRNAs

For each of the 15 miRNAs, target genes we identified: experimentally validated interactions from miRTarBase [38] and TarBase [39]) and in silico predictions from TargetScan 8.0 [40,41] (accessed on 10 October 2025).

Stage 3. Mapping to immune pathways

A separate literature search was performed in the same databases (PubMed, Scopus, Web of Science, Google Scholar) to characterize the major innate and adaptive immune signaling pathways. The following keywords and their combinations were used: “innate immunity”, “adaptive immunity”, “TLR signaling”, “NF-κB pathway”, “JAK-STAT pathway”, as well as the names of specific target genes (e.g., “MyD88”, “TRAF6”, “IRAK1”, “STAT1”, “JAK2”). Studies were included if they met either of the following criteria:

o Described molecular components of immune signaling cascades (receptors, adaptor proteins, transcription factors, negative regulators), their interactions, and physiological outcomes.
o Investigated miRNA-mediated regulation of these pathways, either in the context of HIV infection or in other biological settings.

For each pathway, key regulatory and effector genes were identified. The target genes of each of the 15 miRNAs were then manually mapped to these pathways to determine which miRNAs may modulate specific immune signaling cascades.

Stage 4. Search for experimental evidence for miRNA–mRNA interactions

For each miRNA–target gene pair (e.g., miR-155 and SOCS1), a targeted literature search was performed in the same databases (PubMed, Scopus, Web of Science, and Google Scholar) to identify studies providing direct experimental evidence of interaction. Pairs lacking published experimental evidence for direct binding or functional regulation were classified as “predicted interactions” (based on miRTarBase/TarBase/TargetScan data without experimental validation). Manual curation supplemented the interaction list, as not all experimentally validated cases are captured in existing databases. The absence of experimental confirmation does not indicate the absence of an interaction; it may reflect that the interaction has not yet been investigated.

**Figure 5:**
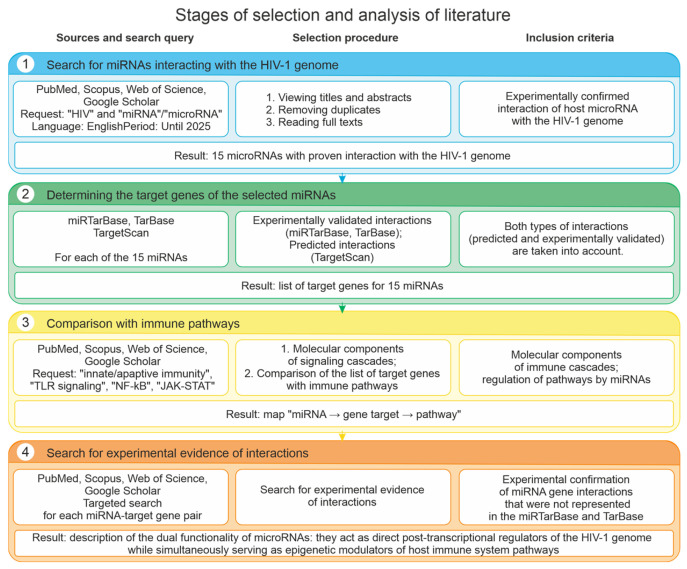
Methodological workflow of the narrative review, depicting the four sequential stages of literature identification, screening, and analysis. Each stage block summarizes the data sources and search terms, the screening procedures and inclusion criteria, and the resulting output.

At each stage, the literature screening and assessment of eligibility were performed by two researchers; discrepancies were resolved by a third. Figure 5 illustrates the sequential stages of the review workflow.

## 5. Conclusions

The analysis conducted within the framework of this narrative review has shown that host miRNAs capable of directly binding to the HIV-1 genome possess dual functionality, which defines their central role in the pathogenesis of infection.

On the one hand, the panel of 15 host miRNAs identified here (miR-29a/b-3p, miR-125b-5p, miR-150-5p, miR-223-3p, miR-28-5p, miR-92a-3p, miR-196b-5p, miR-382-5p, miR-1290, miR-149-5p, miR-133b, miR-138-5p, miR-378a-3p, miR-324-5p) operate as direct post-transcriptional silencers of the HIV-1 genome: (*nef*, *pol*, *vpr*, *gag*, *env*, *vif*) and the 3′-UTR. Such direct antiviral activity supports the therapeutic premise of employing synthetic miRNA mimics to suppress viral replication.

On the other hand, 13 of these miRNAs concurrently modulate key host immune signaling pathways:

- Suppress TLR-mediated recognition (TLR3, TLR7, TLR8, adapters MyD88, TRAF3/6, IRAK1/4);
- Reduce NF-κB activity (REL, RELA, NFKB1), which promotes viral transition to latency;
- Modulate the JAK/STAT signaling axis (STAT1–3, STAT5A/B, and JAK2), thereby perturbing cytokine-driven signaling;
- Target negative feedback regulators of STAT signaling, including members of the SOCS and PIAS protein families;
- Suppress the transcription factors T-bet and Eomes, leading to exhaustion of cytotoxic T lymphocytes and weakened immune surveillance over the reservoir.

Accordingly, the direct antiviral and immunomodulatory activities of these miRNAs are functionally intertwined. Attenuation of host immune signaling pathways facilitates the establishment of viral latency and stabilizes the reservoir, whereas direct targeting of viral transcripts may further compromise immune competence through unintended modulation of host gene expression. Thus, the described miRNA network is consistent with a conceptual model of a self-sustaining proviral regulatory system, as inferred from the synthesis of published data.

Particular attention is warranted for miR-133b and miR-196b-5p. The analysis indicates that these two miRNAs selectively target SOCS and PIAS family members without engaging other immune pathways examined in this study. This specificity positions them as attractive candidates for targeted therapeutic modulation with a potentially reduced risk of off-target consequences.

In practical terms, manipulating the activity of the identified miRNAs via mimics or antagomirs offers a conceptual foundation for two therapeutic approaches:

- “Shock and kill”: the use of antagomirs to neutralize miRNAs that enforce latency, thereby promoting proviral reactivation and subsequent eradication of infected cells;
- “Block and lock”: administration of miRNA mimics targeting NF-κB or JAK/STAT pathways to deepen and stabilize the latent state, thus shielding the reservoir from reactivation stimuli.

In summary, miRNAs that engage the HIV-1 genome serve as pivotal mediators at the virus–host interface. Their capacity to concurrently modulate numerous signaling cascades positions them as central determinants of HIV-1 pathogenesis and as attractive, yet technically demanding, targets for therapeutic strategies pursuing a functional cure.

## Figures and Tables

**Table 1:** 15 miRNAs with experimentally confirmed direct binding to the HIV-1 genome identified.

HIV-1 Target Region	miRNAs Identified	Experimental Method	Evidence Level *	Study (Reference)
* nef *	hsa-miR-29a-3p, hsa-miR-29b-3p	Luciferase assay, qRT-PCR	Functional MTI (strong)	[33]
* vpr *, * env *	hsa-miR-149-5p, hsa-miR-133b, hsa-miR-138-5p, hsa-miR-378a-3p	Bioinformatics + molecular biology	Functional MTI (strong)	[34]
* gag *, * env *, * vif *, * pol *	hsa-miR-149-5p, hsa-miR-133b, hsa-miR-138-5p, hsa-miR-378a-3p, hsa-miR-324-5p, hsa-miR-92a-3p	Bioinformatics + molecular biology	Functional MTI (strong)	[35]
3′-UTR	hsa-miR-28-3p, hsa-miR-125b-5p, hsa-miR-150-5p, hsa-miR-223-3p, hsa-miR-382-5p	Dual-luciferase reporter assay	Functional MTI (strong)	[37]
3′-UTR	hsa-miR-196b-5p, hsa-miR-1290	Dual-luciferase reporter assay	Functional MTI (strong)	[17]

* Evidence levels were assigned according to the miRTarBase classification scheme for miRNA–target interactions (MTI): Functional MTI (Strong)—interactions validated by direct functional assays (e.g., luciferase reporter assays, Western blotting, or qRT-PCR with target expression measurements); Functional MTI (Weak)—interactions identified through high-throughput sequencing of RNA-binding protein immunoprecipitates (HITS-CLIP, PAR-CLIP, CLASH) but lacking direct functional validation of target repression.

**Table 2:** Host miRNA interactions with Toll-like receptor (TLR) target genes.

miRNA	Target Gene	Experimental Method	Evidence Level *	Study (Reference)
hsa-miR-150-5p	* TLR7 *	HITS-CLIP//NGS	Functional MTI (Weak)	[53]
hsa-miR-378-3p	* TLR8 *	—	Predicted only (TargetScan)	
hsa-miR-92a-3p	* TLR3 *	PAR-CLIP//NGS	Functional MTI (Weak)	[54]

* Evidence levels were assigned according to the miRTarBase classification scheme for miRNA–target interactions (MTI): Functional MTI (Strong)—interactions validated by direct functional assays (e.g., luciferase reporter assays, Western blotting, or qRT-PCR with target expression measurements); Functional MTI (Weak)—interactions identified through high-throughput sequencing of RNA-binding protein immunoprecipitates (HITS-CLIP, PAR-CLIP, CLASH) but lacking direct functional validation of target repression.

**Table 3:** MiRNAs involved in the regulation of MyD88-dependent and TRIF-dependent pathways in TLR signaling.

miRNA	Target Gene	Experimental Method	Evidence Level *	Study (Reference)
hsa-miR-149-5p	* MYD88 *	ELISA//qRT-PCR//Western blot	Functional MTI (strong)	[64]
hsa-miR-125b-5p	* TRAF6 *	qRT-PCR	Functional MTI (strong)	[66,68]
hsa-miR-149-5p	* TRAF6 *	Luciferase reporter assay//qRT-PCR//Western blot	Functional MTI (strong)	[65]
hsa-miR-133b	* TRAF3 *	—	Predicted only (TargetScan)	—
hsa-miR-29a-3p	* TRAF3 *	HITS-CLIP	Functional MTI (Weak)	[70,71,72]
hsa-miR-29b-3p	* TRAF3 *	HITS-CLIP	Functional MTI (Weak)	[70,71,72]
hsa-miR-92a-3p	* TRAF3 *	PAR-CLIP//HITS-CLIP	Functional MTI (Weak)	[73]
hsa-miR-378-3p	* TRAF3 *	—		—
hsa-miR-92a-3p	* IRAK1 *	CLASH	Functional MTI (Weak)	[69]
hsa-miR-150-5p	* IRAK4 *	HITS-CLIP	Functional MTI (Weak)	[53]

* Evidence levels were assigned according to the miRTarBase classification scheme for miRNA–target interactions (MTI): Functional MTI (Strong)—interactions validated by direct functional assays (e.g., luciferase reporter assays, Western blotting, or qRT-PCR with target expression measurements); Functional MTI (Weak)—interactions identified through high-throughput sequencing of RNA-binding protein immunoprecipitates (HITS-CLIP, PAR-CLIP, CLASH) but lacking direct functional validation of target repression.

**Table 4:** MiRNAs targeting components of the NF-κB transcription factor complex.

miRNA	Target Gene	Experimental Method	Evidence Level *	Study (Reference)
hsa-miR-150-5p	* REL *	HITS-CLIP	Functional MTI (Weak)	[87]
hsa-miR-29a-3p	* REL *	PAR-CLIP	Functional MTI (Weak)	[86]
hsa-miR-29b-3p	* REL *	PAR-CLIP	Functional MTI (Weak)	[86]
hsa-miR-92a-3p	* REL *	PAR-CLIP	Functional MTI (Weak)	[85]
hsa-miR-138-5p	* RELA *	—	Predicted only (TargetScan)	—
hsa-miR-28-5p	* RELA *	—	Predicted only (TargetScan)	—
hsa-miR-324-5p	* RELA *	CLASH	Functional MTI (Weak)	[69]
hsa-miR-138-5p	* NFKB1 *	Western blot	Functional MTI (Strong)	[88]
hsa-miR-92a-3p	* NFKB1 *	CLASH	Functional MTI (Weak)	[69]

* Evidence levels were assigned according to the miRTarBase classification scheme for miRNA–target interactions (MTI): Functional MTI (Strong)—interactions validated by direct functional assays (e.g., luciferase reporter assays, Western blotting, or qRT-PCR with target expression measurements); Functional MTI (Weak)—interactions identified through high-throughput sequencing of RNA-binding protein immunoprecipitates (HITS-CLIP, PAR-CLIP, CLASH) but lacking direct functional validation of target repression.

**Table 5:** MiRNAs targeting the JAK-STAT pathway.

miRNA	Target Gene	Experimental Method	Evidence Level *	Study (Reference)
hsa-miR-125b-5p	* JAK2 *	Functional MTI (strong)	qRT-PCR//Western blot	[103]
hsa-miR-150-5p	* STAT1 *	Functional MTI (strong)	Luciferase reporter assay//qRT-PCR//Western blot	[99,100]
hsa-miR-223-3p	* STAT1 *	Functional MTI (strong)	Luciferase reporter assay//qRT-PCR//Western blot	[100]
hsa-miR-92a-3p	* STAT2 *	Functional MTI (Weak)	PAR-CLIP//CLASH	[69,104]
hsa-miR-125b-5p	* STAT3 *	Functional MTI (strong)	Western blot//Luciferase reporter assay//Western blot//Microarray	[105,106,107,108,109]
hsa-miR-223-3p	* STAT3 *	Functional MTI (strong)	qRT-PCR//Western blot	[110]
hsa-miR-29a-3p	* STAT3 *	Functional MTI (strong)	Luciferase reporter assay//qRT-PCR//Western blot	[111,112]
hsa-miR-29b-3p	* STAT3 *	Functional MTI (strong)	Immunofluorescence//Luciferase reporter assay//qRT-PCR//Western blot | Luciferase reporter assay//qRT-PCR//Western blot//Immunohistochemistry	[113,114]
hsa-miR-92a-3p	* STAT3 *	Functional MTI (strong)	Luciferase reporter assay//qRT-PCR//Western blot//CLASH	[69,115]
hsa-miR-223-3p	* STAT5A *		Luciferase reporter assay//qRT-PCR	[116]
hsa-miR-150-5p	* STAT5B *	Functional MTI (strong)	Luciferase reporter assay//qRT-PCR//Western blot	[117]
hsa-miR-28-5p	* STAT5B *	Functional MTI (strong)	Luciferase reporter assay	[118]

* Evidence levels were assigned according to the miRTarBase classification scheme for miRNA–target interactions (MTI): Functional MTI (Strong)—interactions validated by direct functional assays (e.g., luciferase reporter assays, Western blotting, or qRT-PCR with target expression measurements); Functional MTI (Weak)—interactions identified through high-throughput sequencing of RNA-binding protein immunoprecipitates (HITS-CLIP, PAR-CLIP, CLASH) but lacking direct functional validation of target repression.

**Table 6:** MiRNAs targeting STAT inhibitors.

miRNA	Target Gene	Experimental Method	Evidence Level *	Study (Reference)
hsa-miR-133b	* SOCS2 *	—	Predicted only (TargetScan)	
hsa-miR-196b-5p	* SOCS2 *	—	Predicted only (TargetScan)	
hsa-miR-125b-5p	* SOCS4 *	Luciferase reporter assay	Functional MTI (strong)	[134]
hsa-miR-196b-5p	* SOCS4 *	—	Predicted only (TargetScan)	
hsa-miR-150-5p	* SOCS5 *	PAR-CLIP	Functional MTI (Weak)	[137]
hsa-miR-92a-3p	* SOCS5 *	Luciferase reporter assay//qRT-PCR//Western blot	Functional MTI (strong)	[136]
hsa-miR-138-5p	* SOCS6 *	—	Predicted only (TargetScan)	
hsa-miR-149-5p	* SOCS6 *	—	Predicted only (TargetScan)	
hsa-miR-92a-3p	* SOCS6 *	—	Predicted only (TargetScan)	
hsa-miR-29a-3p	* SOCS7 *	—	Predicted only (TargetScan)	
hsa-miR-29b-3p	* SOCS7 *	—	Predicted only (TargetScan)	
hsa-miR-133b	* PIAS2 *	HITS-CLIP	Functional MTI (Weak)	[139]
hsa-miR-150-5p	* PIAS2 *	HITS-CLIP	Functional MTI (Weak)	[138]
hsa-miR-125b-5p	* PIAS3 *	Luciferase reporter assay//Western blot	Functional MTI (strong)	[135]
hsa-miR-29a-3p	* PIAS4 *	—	Predicted only (TargetScan)	
hsa-miR-29b-3p	* PIAS4 *	—	Predicted only (TargetScan)	
hsa-miR-324-5p	* PIAS4 *	CLASH	Functional MTI (Weak)	[69]
hsa-miR-92a-3p	* PIAS4 *	—	Predicted only (TargetScan)	

* Evidence levels were assigned according to the miRTarBase classification scheme for miRNA–target interactions (MTI): Functional MTI (Strong)—interactions validated by direct functional assays (e.g., luciferase reporter assays, Western blotting, or qRT-PCR with target expression measurements); Functional MTI (Weak)—interactions identified through high-throughput sequencing of RNA-binding protein immunoprecipitates (HITS-CLIP, PAR-CLIP, CLASH) but lacking direct functional validation of target repression.

**Table 7:** MiRNAs targeting Eomes and T-bet (Tbx21).

miRNA	Target Gene	Experimental Method	Evidence Level *	Study (Reference)
hsa-miR-29a-3p	* EOMES *	—	Predicted only (TargetScan)	—
hsa-miR-29b-3p	* EOMES *	—	Predicted only (TargetScan)	—
hsa-miR-92a-3p	* EOMES *	HITS-CLIP	Functional MTI (Weak)	[152]
hsa-miR-29a-3p	* TBX21 *	—	Predicted only (TargetScan)	—
hsa-miR-29b-3p	* TBX21 *	Luciferase reporter assay	Functional MTI (strong)	[151]

* Evidence levels were assigned according to the miRTarBase classification scheme for miRNA–target interactions (MTI): Functional MTI (Strong)—interactions validated by direct functional assays (e.g., luciferase reporter assays, Western blotting, or qRT-PCR with target expression measurements); Functional MTI (Weak)—interactions identified through high-throughput sequencing of RNA-binding protein immunoprecipitates (HITS-CLIP, PAR-CLIP, CLASH) but lacking direct functional validation of target repression.

**Table 8:** Predicted biological consequences of “shock and kill” and “block and lock” therapeutic strategies for miRNAs with experimentally confirmed dual activity in HIV-1 infection.

miRNA	Preferred Strategy	Rationale	Proposed Direction of Action
miR-92a-3p	Block and Lock	Suppression of TLR3 reduces innate recognition of HIV RNA. Suppression of REL and NFKB1 attenuates NF-κB activity, reducing LTR-directed transcription and promoting latency. Suppression of STAT2/STAT3 dampens cytokine-driven reactivation signals. Suppression of SOCS5 may enhance JAK/STAT signaling. Direct suppression of *gag*, *env*, *vif*, and *pol* provides additional antiviral activity.	Upregulation (miRNA mimic). Delivery of a miR-92a-3p mimic will attenuate TLR3-mediated innate recognition of HIV RNA, reduce NF-κB-driven LTR transcription, dampen cytokine-mediated reactivation signals, and directly restrict translation of gag, env, vif, and pol, thereby reinforcing and maintaining HIV latency.
miR-150-5p	Block and Lock	Suppression of TLR7 and IRAK4 blocks two nodes of the MyD88-dependent cascade, reducing innate immune activation. Suppression of REL promotes proviral silencing. Suppression of STAT1 reduces interferon-stimulated antiviral responses. Suppression of STAT5B potentially reduces viral reservoir size. Suppression of SOCS5 and PIAS2 may cause compensatory JAK/STAT hyperactivation. Direct suppression of the 3′-UTR restricts viral translation in resting CD4+ T cells.	Upregulation (miRNA mimic). Delivery of a miR-150-5p mimic will block two nodes of the MyD88-dependent innate immune cascade, reduce proviral reactivation signals, and directly restrict viral translation in resting CD4+ T cells, consolidating latency and potentially reducing the size of the viral reservoir.
miR-125b-5p	Block and Lock	Suppression of TRAF6 blocks a central node of the MyD88-dependent cascade, reducing NF-κB activation. Suppression of JAK2 limits cytokine-driven STAT activation. Suppression of STAT3 reduces survival signals in infected cells and dampens cytokine-mediated immune activation. Suppression of SOCS4 and PIAS3 disrupts negative feedback on JAK/STAT. Direct suppression of the 3′-UTR restricts viral translation in resting CD4+ T cells.	Upregulation (miRNA mimic). Delivery of a miR-125b-5p mimic will attenuate NF-κB activation, limit cytokine-driven STAT signaling, reduce survival signals in infected cells, and directly restrict viral translation in resting CD4+ T cells, promoting and sustaining HIV latency.
miR-29b-3p	Shock and Kill	De-repression of TRAF3 restores TRIF-dependent IRF3 activation and IFN-β production. De-repression of REL activates NF-κB and stimulates proviral reactivation. De-repression of STAT3 enhances cytokine-driven T-cell activation. De-repression of TBX21 restores T-bet expression, enhancing Th1/Tc1 differentiation and cytotoxic CD8+ T-cell function. Restored T-bet improves immune clearance of reactivated infected cells. Direct de-repression of *nef* accelerates replication of reactivated virus. This strategy requires concomitant ART.	Downregulation (antagomir/inhibitor). Administration of a miR-29b-3p antagomir will restore TRIF-dependent IFN-β production, reactivate NF-κB-driven proviral transcription, enhance cytokine-mediated T-cell activation, restore cytotoxic CD8^+^ T-cell function via T-bet, and accelerate replication of reactivated virus, enabling immune-mediated clearance of infected cells. Concomitant ART is required.
miR-29a-3p	Shock and Kill	De-repression of TRAF3 restores TRIF-dependent IRF3 activation and IFN-β production. De-repression of REL activates NF-κB and stimulates proviral reactivation. De-repression of STAT3 enhances cytokine-driven T-cell activation. Unlike miR-29b-3p, for which no experimentally confirmed interaction with TBX21 has been identified, restoration of cytotoxic CD8+ T-cell function is less predictable. Direct de-repression of *nef* accelerates replication of reactivated virus. This strategy requires concomitant ART.	Downregulation (antagomir/inhibitor). Administration of a miR-29a-3p antagomir will restore TRIF-dependent IFN-β production, reactivate NF-κB-driven proviral transcription, enhance cytokine-mediated T-cell activation, and accelerate replication of reactivated virus, facilitating immune-mediated clearance of infected cells. Concomitant ART is required.
miR-223-3p	Block and Lock	Suppression of STAT1 reduces interferon-stimulated antiviral responses. Suppression of STAT3 dampens reactivation signals. Suppression of STAT5A disrupts IL-2-dependent maintenance of CD4+ T-cell memory, potentially reducing viral reservoir size. Direct suppression of the 3′-UTR restricts viral translation in resting CD4+ T cells. Plasma levels of miR-223-3p correlate with viral load and CD4+ T-cell counts, enabling use as a monitoring biomarker during therapy.	Upregulation (miRNA mimic). Delivery of a miR-223-3p mimic will reduce interferon-stimulated antiviral signaling, dampen cytokine-driven reactivation, disrupt IL-2-dependent maintenance of the CD4^+^ T-cell memory compartment, and directly restrict viral translation in resting CD4^+^ T cells, supporting latency maintenance.
miR-149-5p	Block and Lock	Suppression of MyD88 and TRAF6 blocks two key nodes of the MyD88-dependent cascade, attenuating innate immune activation. Direct suppression of five viral genes provides the broad direct antiviral coverage.	Upregulation (miRNA mimic). Delivery of a miR-149-5p mimic will block two key nodes of the MyD88-dependent innate immune cascade and directly restrict translation of five viral genes, providing broad attenuation of immune activation and antiviral coverage to promote latency.
miR-138-5p	Block and Lock	Suppression of NFKB1 promotes proviral silencing. Direct suppression of five viral genes provides broad direct antiviral coverage.	Upregulation (miRNA mimic). Delivery of a miR-138-5p mimic will promote proviral silencing by attenuating NF-κB activity and directly restricting translation of five viral genes, providing broad antiviral coverage to consolidate latency.
miR-324-5p	Block and Lock	Suppression of RELA promotes proviral silencing. Suppression of PIAS4 disrupts the inactivation of STAT factors, which may cause compensatory JAK/STAT hyperactivation. Direct suppression of *env* and *vpr* limits the synthesis of the envelope protein and Vpr functions.	Upregulation (miRNA mimic). Delivery of a miR-324-5p mimic will promote proviral silencing by attenuating NF-κB/RELA-driven transcription and directly restricting synthesis of Env and Vpr, limiting viral replication capacity and supporting latency maintenance.
miR-133b	Block and Lock	Suppression of PIAS2 disrupts the inactivation of STAT1/STAT4, which may enhance JAK/STAT signaling and interferon responses. Direct suppression of *env* and *vpr* limits the synthesis of the envelope protein and Vpr functions.	Upregulation (miRNA mimic). Delivery of a miR-133b mimic will enhance JAK/STAT signaling and interferon responses by disrupting negative feedback on STAT1/STAT4, and directly restrict synthesis of Env and Vpr, limiting viral replication capacity and supporting latency maintenance.
miR-28-5p	Block and Lock	Suppression of STAT5B disrupts the maintenance of memory CD4+ T cells and regulatory T cells, reducing survival signals that sustain the reservoir. Direct suppression of the 3′-UTR restricts viral translation in resting CD4+ T cells.	Upregulation (miRNA mimic). Delivery of a miR-28-5p mimic will reduce survival signals sustaining memory CD4^+^ T cells and regulatory T cells within the viral reservoir and directly restrict viral translation in resting CD4^+^ T cells, thereby reducing reservoir size and promoting durable latency.

## Data Availability

The raw data supporting the conclusions of this article will be made available by the authors upon request.

## Abbreviations

The following abbreviations are used in this manuscript:

3′-UTR	3′-untranslated region
AP-1	Activator protein-1
JNK	C-Jun N-terminal kinase
CLASH	Cross-linking, ligation, and sequencing of hybrids
CTLs	Cytotoxic T lymphocytes
dsRNA	Double-stranded RNA
ERK	Extracellular signal-regulated kinase
HITS-CLIP	High-throughput sequencing of RNA isolated by crosslinking immunoprecipitation
ISGs	IFN-stimulated genes
IRAK	Interleukin-1 receptor-associated kinase
IRF	Interferon regulatory factor
JAK	Janus kinase
LTR	Long terminal repeat
MAPK	Mitogen-activated protein kinase
MHC	Major histocompatibility complex
MAP3K7	Mitogen-activated protein kinase kinase kinase 7
NF-κB	Nuclear factor κB
PAR-CLIP	Photoactivatable ribonucleoside-enhanced crosslinking and immunoprecipitation
PAMP	Pathogen-associated molecular pattern
PRR	Pattern recognition receptor
PIAS	Protein inhibitors of activated STAT
PTP	Protein tyrosine phosphatase
ssRNA	Single-stranded RNA
STAT	Signal transducer and activator of transcription
SOCS	Suppressor of cytokine signaling
TAK1	TGF-β-activated kinase 1
TLR	Toll-like receptor
TRAF6	TNF receptor-associated factor 6
